# Advancing Medical Professionalism in US Military Detainee Treatment

**DOI:** 10.1371/journal.pmed.1001930

**Published:** 2016-01-05

**Authors:** Leonard S. Rubenstein, Scott A. Allen, Phyllis A. Guze

**Affiliations:** 1 Center for Public Health and Human Rights, Johns Hopkins Bloomberg School of Public Health, Baltimore, Maryland, United States of America; 2 Johns Hopkins Berman Institute of Bioethics, Baltimore, Maryland, United States of America; 3 University of California at Riverside School of Medicine, Riverside, California, United States of America

## Abstract

Leonard Rubenstein and colleagues argue that professional associations should ensure that military rules do not require health professionals to choose between service to their country and ethical practice.

**Summary Points**

- The United States Department of Defense and Central Intelligence Agency (CIA) promulgated policies and requirements that required health professionals to participate in the mistreatment of counter-terrorism detainees through participation in such practices as abusive interrogation and force-feeding of detainees, in violation of ethical standards established by associations representing the health professions.
- A report of the Defense Health Board to the Secretary of Defense on military medical ethics released in 2015 found that the Department of Defense “does not have an enterprise-wide, formal, integrated infrastructure to systematically build, support, sustain, and promote an evolving ethical culture within the military health care environment.”
- The Board also found that ethical codes promulgated by the health professions, including the duty to avoid harm, provide a sound basis for military medical practice, even taking into account the unique challenges often faced by military health professionals in reconciling the military mission with patient needs.
- The health professional community should urge the Secretary of Defense to adopt and implement the recommendations of the Defense Health Board, rescind directives authorizing participation of health professionals in interrogation and force-feeding because they are inconsistent with professional ethics, and provide ongoing advice and support for the reform process.

Principles of ethical conduct are essential to clinical practice and the social legitimacy of the health professions. The professions derive the obligations of health professionals from moral principles, the traditions of medicine, and a social contract with society. They reflect these obligations in codes adopted by professional organizations. As the Defense Health Board, an independent Federal Advisory Committee to the Secretary of Defense, recently recognized in a review of medical ethics in the United States military, health professionals in the armed services must adhere to these professional obligations even as they face responsibilities to accomplish the military mission [1]. The Board’s affirmation of these obligations, most notably first loyalty to the patient ([1]; [2], Principle VIII and Opinion 10.015; [3], Principal A and 3.04; [4], Provision 2 and section 2.1; [5]), along with its recommendations for structural reform in military medical ethics, has implications for health professional participation in intelligence and security functions in connection with counter-terrorism detainees. Adopting its recommendations would restore the integrity of military medical ethics and require rescission of requirements that health professionals participate in interrogation and force-feeding of detainees. It is incumbent on the profession to support the Board’s recommendations and urge the Secretary of Defense to adopt them.

## Erosion of Medical Ethics in Detainee Treatment after 9/11

A wealth of evidence shows that the Department of Defense (DoD) and Central Intelligence Agency (CIA) physicians, psychologists, and nurses violated their professional obligations, including duties of beneficence, non-maleficence, and respect for patient autonomy, in the service of brutal detention and interrogation practices. We know, for example, that during interrogations involving the use of long-term sleep deprivation, shackling, sexual humiliation, isolation, and other forms of torture, military physicians intervened to allow torture to continue [6–9]. The Executive Summary of the report of the Senate Select Committee on Intelligence on post-9/11 CIA detention programs, declassified and released in late 2014, shows in detail how CIA physicians and other health professionals facilitated and approved shackling naked detainees in a standing position for as long as a week to deprive them of sleep, oversaw the dousing of detainees with water close to freezing temperatures, supervised the use of waterboarding and engaged in “rectal feeding” [10], which has no medical basis.

A comprehensive review by an independent task force convened by the Institute on Medicine as a Profession, composed of 20 experts in bioethics, military medicine, and human rights, examined the conduct of medical personnel in military and intelligence detention facilities and found that medical participation in torture was not the product of rogue individuals ([6], finding 3). Rather, it found that the Department of Defense and the CIA “*required* physicians, psychologists, and other health professionals to act contrary to their professional obligations” (emphasis added). According to the task force, the agencies directed them to approve methods of torture, monitor interrogations involving torture and other abuses, suggest harsh methods of interrogation in individual cases, set conditions of confinement that would be most disruptive to that individual’s sense of well-being, and forcibly feed hunger strikers, all in violation of US and international medical ethical standards [6].

Neither the US military nor the CIA has ever claimed that military necessity or national security considerations justified setting aside physicians’ ethical obligations. Instead, contrary to military tradition and the authority of the profession to set expectations for ethical conduct, the agencies reinterpreted and rewrote ethical standards in a manner designed to facilitate the participation of physicians and other health professionals in torture and cruel, inhuman, and degrading treatment. The departures from ethical traditions have three features. First, the obligation to avoid harm was severely restricted. The Department of Defense instructed physicians and psychologists not to adhere to the ethic of beneficence and non-maleficence so long as they were assigned to tasks other than patient care, particularly interrogation, even though they were still being asked to employ their medical expertise [11]. As a result, the directives ordered certain physicians and psychologists involved in interrogation to engage in “psychological assessments of the character, personality, social interactions, and other behavioral characteristics of detainees and to advise” interrogators about them [12]. Implementation guidelines advised them to “evaluate the psychological strengths and vulnerabilities of detainees, and to assist in integrating these factors into a successful interrogation/debriefing process” [13]. The CIA equated the obligation to do no harm to mean only to “prevent severe physical or mental pain or suffering” [14], allowing health professionals to inflict any harm that, in its view, did not constitute torture.

Second, in determining the nature of professional obligations, both the CIA and Department of Defense reframed the duty of non-maleficence into a duty only to obey the law, such that participating in harm short of torture or cruel, inhuman, or degrading treatment became permissible [6,11,14,15].

Third, military physicians have been instructed that they can violate patient autonomy and traditional clinical loyalties to patients in order to facilitate force-feeding of hunger strikers, including through the use of restraint chairs [11,16], a practice that is never ethically acceptable and amounts to inhuman and degrading treatment [17,18].

The CIA has closed secret detention facilities, known as Black Sites, but the military directives and practices remain in effect at Guantanamo Bay [11,12].

## The Response of the Medical Profession and the Defense Health Board

Medical groups responded to these directives by affirming and further clarifying ethical obligations [19,20]. They all reinforced that there is no legitimate role for physicians in monitoring or participating in any way in individual interrogations, no matter what their role, even in lawful interrogation methods; there exists no room in medical practice to inflict any harm or pain on a patient for non-clinical reasons. Among health professional organizations, only the American Psychological Association took a permissible view of participation [21,22]. The American Medical Association (AMA) also reaffirmed that physicians must never force-feed a competent hunger striker to break his protest [23]. The only appropriate, ethical roles of the physician caring for a hunger striker is to assess whether the patient is on a hunger strike, determine the capacity of the patient to make informed health decisions, counsel the patient regarding the risks and benefits of various decisions, and provide medical care with the patient’s consent [17].

The Department of Defense declined to change any of its directives or practices. In May 2011, however, the Assistant Secretary of Defense for Health Affairs requested that the Secretary of Defense ask the Defense Health Board to review challenges stemming from military health professionals’ dual roles as military officers and medical providers. In January 2013, the Under Secretary of Defense formally requested the Board’s advice on two questions: First, how can military professionals appropriately balance their obligations to patients against obligations as military officers to help commanders maintain military readiness? And second, how much latitude should military medical professionals be given to refuse participation in medical procedures or request excusal from military operations with which they have ethical reservations or disagreement [1]? The Board assigned the task to its Medical Ethics Subcommittee.

The Subcommittee expanded its review far beyond the request from the Under Secretary, examining the foundations of military medical ethics, the nature of ethical conflicts arising in military health practices, such as fitness-for-duty exams, battlefield triage, and detainee treatment, and the Department of Defense’s guidelines, training, and structures supporting health professional ethics. Furthermore, it focused on institutional responsibility rather than individual decision-making. After consultations with military personnel and outside experts and internal deliberations, the Subcommittee presented its report and recommendations to the Board in February 2015. The Board adopted all of them, making only technical corrections.

The Board recognized that military physicians, like those in other complex environments, often face “dual loyalty” conflicts between ethical duties and organizational requirements [24]. It concluded, however, that codes established by medical professional organizations “are consistent with and applicable to much of the health care practiced by military personnel in the [Military Health System (MHS)]” ([1], finding 2) and urged, “Throughout its policies, guidance, and instructions, DoD must ensure that the military health care professional’s first ethical obligation is to the patient” ([1], recommendation 2). More broadly, it found that “DoD does not have an enterprise-wide, formal, integrated infrastructure to systematically build, support, sustain, and promote an evolving ethical culture within the military health care environment” ([1], finding 1). It therefore called for mechanisms to foster such an environment and respect for key ethical obligations of health personnel. One key step, it said, was to establish a code of conduct for military health professionals “based on accepted codes from various health care professions to serve as a guidepost to promote ethical leadership and set a standard for the cultural ethos of the MHS” ([1], recommendation 4). Such a code, it said, should be reviewed regularly to assure it remains consistent with those of professional medical organizations.

The Board also made a series of recommendations designed to strengthen the infrastructure of medical ethics in the military and training and support of health professionals. These include assuring mandatory training (including pre-deployment) for military health professionals; improving existing ethics courses; creating mechanisms for support, consultation, and debriefing of health professionals facing ethical challenges, including an online ethics portal; and inclusion of senior medical officers on commanders’ staff. It also recommended tightened standards of medical confidentiality and enhanced protection for military physicians confronted by demands from commanders to breach professional obligations: “DoD leadership, particularly the line commands, should excuse health care professionals from performing medical procedures that violate their professional code of ethics, State medical board standards of conduct, or the core tenets of their religious or moral beliefs” ([1], recommendation 3). Adopting such a policy would protect health professionals who seek to act ethically in the face of demands to elevate military goals over patient interests, as in the case of a Navy nurse who refused to participate in force-feeding at Guantanamo Bay and was threatened with prosecution and discharge. Protests by the American Nurses Associations and human rights organizations led the Navy to relent, but only after almost a year where the threat of prosecution and discharge hung over him [25].

There were, nevertheless, some limitations in the Board’s recommendations. Most notably, it did not address the inconsistency between Department of Defense directives for health professional involvement in hunger strikes and interrogation and the principles it cited. In our view, the Board’s failure to call for a prohibition of any military health professional involvement in interrogation or force-feeding hunger strikers in its recommendations is a serious deficiency. It should also have recommended transparency and oversight in the reform process.

## Moving Forward with Reform

Notwithstanding their limitations, the recommendations by the Board warrant support of the medical community and adoption and implementation by the Secretary of Defense as a means of enabling military health professionals who interact with detainees to act in accordance with the ethical obligations of their profession. The need for action is reinforced by the 2015 independent investigation showing American Psychological Association (APA) collusion with the Department of Defense in approving the participation of psychologists in counter-terrorism interrogations, and in masking the abuses that flowed from it [22]. After the investigation’s revelations of the organization’s improper conduct, the APA changed its position, and now prohibits psychologists from participating in national security interrogations [26]. As a result, no professional organization finds an ethical basis for health professional participation in interrogation or for applying different ethical standards based on the professional’s role.

As part of their task to stand for the moral values underlying health practice, health professional organizations should urge adoption of the Board’s recommendations and actively participate in the reform process toward ending the manipulations of ethics and military medical personnel that contributed to detainee abuse. In the last decade, health professional associations, while criticizing directives requiring breaches of ethical obligations in the treatment of detainees, have not been as active as they could be in protecting professional integrity in military medical practice. The engagement of the American Nurses Association (ANA) in defending the Navy nurse under threat of discipline for refusing to force-feed provides a promising exception. The Association was outspoken in demanding that he not be punished for adhering to nursing ethics [27].

The work, however, needs to go beyond defense of individual service members. Professional associations of physicians and psychologists should call for implementation of the Board’s recommendations, as the ANA, along with the Society of General Internal Medicine, did [27,28] and for rescission of policies that require health professionals to participate in interrogation and force-feeding. They should provide ongoing consultation as the military adopts a code of ethics consistent with professional values and support structures. By example, that stance could also promote respect for medical ethics in the CIA. Finally, professional associations should seek congressional oversight of the reform process as a means for ensuring integrity in military medical ethics.

Health professionals who have volunteered to serve for their country should never be asked or directed to violate their professional ethics, nor be allowed to be instruments in detainee abuse. To be true to their role in setting ethical standards for practice and creating an environment that enables health professionals to follow them, professional associations must do everything in their power to make sure that military rules do not require health professionals to choose between service to their country and ethical practice.

## Abbreviations

AMA: American Medical Association
ANA: American Nurses Association
APA: American Psychological Association
CIA: Central Intelligence Agency
DoD: Department of Defense
MHS: Military Health System
